# Cerebrovascular reactivity in Alzheimer's disease signature regions is associated with mild cognitive impairment in adults with hypertension

**DOI:** 10.1002/alz.13572

**Published:** 2023-12-18

**Authors:** Vahan Aslanyan, Wendy J. Mack, Nancy E. Ortega, Ilya M. Nasrallah, Nicholas M. Pajewski, Jeff D. Williamson, Judy Pa

**Affiliations:** ^1^ Department of Population and Public Health Sciences Keck School of Medicine University of Southern California Los Angeles California USA; ^2^ Alzheimer's Disease Cooperative Study (ADCS) Department of Neurosciences University of California, San Diego La Jolla California USA; ^3^ Department of Radiology University of Pennsylvania Philadelphia Pennsylvania USA; ^4^ Department of Biostatistics and Data Science Division of Public Health Science Wake Forest University School of Medicine Winston‐Salem North Carolina USA; ^5^ Section of Gerontology and Geriatric Medicine Department of Internal Medicine Wake Forest University School of Medicine Winston‐Salem North Carolina USA

**Keywords:** biomarkers, cerebrovascular reactivity, cognitive dysfunction, hypertension, mild cognitive impairment

## Abstract

**INTRODUCTION:**

Vascular risk factors contribute to cognitive decline suggesting that maintaining cerebrovascular health could reduce dementia risk. The objective of this study is to evaluate the association of cerebrovascular reactivity (CVR), a measure of brain blood vessel elasticity, with mild cognitive impairment (MCI) and dementia.

**METHODS:**

Participants were enrolled in the Systolic Blood Pressure Intervention Trial Memory and Cognition in Decreased Hypertension (SPRINT‐MIND) magnetic resonance imaging substudy. Baseline CVR in Alzheimer's disease (AD) signature regions were primary variables of interest. The occipital pole and postcentral gyrus were included as control regions.

**RESULTS:**

Higher AD composite CVR was associated with lower MCI risk. No significant associations between inferior temporal gyrus, occipital pole, or postcentral gyrus CVR and MCI risk, or any regional CVR–combined risk associations were observed.

**DISCUSSION:**

CVR in AD signature regions is negatively associated with occurrence of MCI, implicating CVR in AD signature regions as a potential mechanism leading to cognitive impairment.

## 1 BACKGROUND

Dementia is a complex neurological condition often caused by neurodegenerative pathologies and exacerbated by cerebrovascular diseases. Dementia can be characterized by gradual progression from normal cognition to mild cognitive impairment (MCI) and then dementia. Alzheimer's disease (AD) pathology and cerebrovascular risk factors, such as hypertension, high cholesterol, diabetes, and stroke, contribute to the risk of dementia through multiple possible pathways,^1^, ^2^, ^3^ including impaired regional cerebrovascular reactivity (CVR),^4^ a measure of cerebrovascular responsiveness.

Studies have shown that persons with AD, the most prevalent pathology in adults with dementia (65.3%),^5^ and MCI have lower CVR compared to healthy older adults,^6^, ^7^, ^8^ including a link between lower CVR and poorer cognitive performance.^9^, ^10^ Similarly, transgenic mice overproducing amyloid and transforming growth factor (TGF)‐β1 developed an early progressive decline of cerebral arterial function and displayed arterial dysfunction unrelated to oxidative stress. While there is evidence that chronic exposure to increased amyloid beta (Aβ) causes microvascular dysfunction before development of cerebral amyloid angiopathy (CAA) in amyloid‐overproducing mice,^11^ independent of plaque accumulation, neurovascular changes occurred in parallel to age‐modulated accumulation of amyloid plaques, indicating a correlation between decreased CVR and increased AD‐like neuropathological changes.^12^, ^13^

Findings from the Religious Orders Study and the Memory and Aging Project (ROSMAP) relate moderate and severe CAA (prevalence of 35.8%), atherosclerosis (33.2% prevalence), and arteriolosclerosis (31.3% prevalence) with cognitive decline.^5^ This supports the notion that neurodegenerative and cerebrovascular pathologies interact to drive cognitive impairment.^14^ Rodent models and human studies have shown that CAA may reduce CVR,^15^, ^16^ possibly through amyloid accumulation in brain vessels, resulting in loss of vessel integrity or loss of blood supply and ischemia.^14^ Older adults with higher carotid intimal medial thickness had a 2.5‐fold increased risk of dementia, solidified by an estimated 2‐fold increased risk among individuals with bilateral plaque.^17^ In two studies, CVR increased after carotid endarterectomy, indicating a close relationship between atherosclerosis and CVR.^18^, ^19^ Similar to findings among participants with atherosclerosis, arteriosclerosis was associated with worse global cognition,^20^ and 1.2‐fold increase in risk of AD.^21^ CVR is a risk marker for incident symptomatic lacunar infarction, usually caused by arteriosclerosis, highlighting its role in cerebrovascular degeneration and potential implication in cognitive decline. While converging evidence implicates CVR as a correlate of cognitive decline, there is limited evidence elucidating the association of CVR and the development of MCI and dementia.

Imaging studies have shown that vascular contractility issues are spatially similar to amyloid imaging findings.^14^, ^22^ Disrupted perivascular amyloid clearance seems to be a pathogenic mechanism in AD and CAA,^14^ suggesting that deteriorating CVR might be an early indicator for progression to dementia. Among middle‐aged adults, increased cardiovascular risk profile was associated with lower CVR in regions underlying the default mode network,^23^ which is implicated in risk of MCI and AD, with some specificity to the hippocampus,^4^ a memory processing hub closely related to AD progression. These indicate a potential relationship between CVR of AD signature regions and subsequent cognitive decline. AD signature regions are the regions closely associated with AD progression, and commonly include the hippocampus, parahippocampus, precuneus, fusiform gyrus (FG), middle temporal gyrus (MTG), inferior temporal gyrus (ITG), and angular gyrus (AG).^24^, ^25^, ^26^

The objective of this study is to investigate the relationship between CVR and MCI. We hypothesize that higher CVR in AD signature regions will be associated with lower rates of adjudication with MCI during the follow‐up. To demonstrate that these associations are specific to AD signature regions, CVR–MCI risk relationships were assessed within the control regions of occipital pole (OP) and postcentral gyrus (PCG), regions typically not affected by neuropathological changes during early stages of AD.^23^ To investigate the specificity of CVR as a predictor of MCI progression, exploratory analyses evaluated the relationships with dementia and a composite outcome of MCI and dementia.

**RESEARCH IN CONTEXT**

1. **Systematic review**: The authors reviewed the literature using traditional sources. Relevant publications describing the rationale and methodology of the current study as well as studies interpreting and contextualizing findings of the current study are appropriately cited.
2. **Interpretation**: The current study establishes a relationship between cerebrovascular reactivity in Alzheimer's disease (AD) signature regions and cognitive decline, characterized by adjudicated mild cognitive impairment, reaffirming the role of CVR and vascular dysfunction in dementia etiology. Identification and treatment of lower CVR might prevent or delay cognitive decline.
3. **Future directions**: CVR could point to new biological pathways that may play an early role in dementia progression. Our findings need to be replicated in a larger cohort and for a longer follow‐up period, allowing for a greater power to detect potential relationship with incident dementia.

## 2 METHODS

### 2.1 Participants

Study participants were selected from the Systolic Blood Pressure Intervention Trial (SPRINT) Memory and Cognition in Decreased Hypertension (SPRINT‐MIND) study magnetic resonance imaging (MRI) substudy. SPRINT‐MIND was a multicenter randomized controlled trial testing the efficacy of intensive blood pressure control on cardiovascular, renal, and cognitive outcomes. Trial design and methods have been described previously.^3^, ^27^, ^28^, ^29^ Trial participants were at least 50 years old, had hypertension, had at least one additional risk factor for heart disease, but did not have diabetes or history of stroke. Additionally, persons residing in nursing homes, diagnosed with dementia, or treated with medication designed for dementia were excluded from the study. Participants were randomized to systolic blood pressure goal of < 120 mmHg (high intensity) or < 140 mmHg (low intensity). As a part of the study, eligible participants with access to the MRI sites were enrolled in the MRI study.^30^ Everyone with available MRI was included in the current study. The trial and the MRI substudy were approved by the institutional review board at participating centers and all participants provided written informed consent.

### 2.2 MRI measures and processing

Baseline MRI scans were used in the current study. MRI collection and processing techniques have been previously described.^28^, ^30^ In summary, structural MRI of the brain included 1 mm isotropic T1 magnetization‐prepared rapid acquisition gradient echo, T2, and T2 fluid‐attenuated inversion recovery images. Participants also underwent a breath‐hold blood oxygen‐level–dependent (BOLD) functional MRI (fMRI) sequence, in which imaging occurred during a block of four 16 second breath holds interspersed with free breathing (repetition time = 2000 ms, echo time = 25 ms, field of view = 224 mm, thickness = 3.5 mm, slices = 35, native resolution = 3.5 mm isotropic). A multi‐atlas, multi‐warp label‐fusion method (MUSE)^31^ was used to segment T1 scans into 145 anatomic regions of interest (ROI). CVR was derived from the breath‐hold BOLD sequence.^30^ Breath holding elevated blood CO_2_ and induced vasodilation to change the BOLD signal. The resulting images were motion corrected and smoothed, and a whole brain generalized linear model analysis was performed. The CVR brain map was then registered to T1 space and interpolated to 1 mm isotropic resolution.^28^, ^30^, ^32^ To reduce participant burden and discomfort, end tidal CO_2_ was not measured.

### 2.3 Regions of interest

AD signature regions were selected based on studies of cortical thinning and volume loss related to AD severity.^24^, ^25^, ^26^ These regions were identified in the MUSE atlas^31^ used for CVR quantification. Hippocampus, parahippocampus, precuneus, FG, MTG, ITG, and AG were selected for the current study based on their regional association with AD pathologies and availability in the CVR atlas. An AD composite CVR was created as a volume‐weighted sum of CVRs of AD signature regions. Additionally, the OP and the PCG were selected as reference regions because they demonstrate atrophy at late stages of AD progression.^33^ The PCG, a region in the sensorimotor cortex, is supplied by the anterior circulation like most components of the AD signature region, while OP is supplied by the posterior circulation, along with portions of the hippocampus and medial temporal lobe.

### 2.4 Plasma biomarkers

Blood collection and plasma biomarker processing have been previously described.^34^ Biomarkers were processed for participants that were ≥ 60 years at the time of randomization due to available resources and potential to observe more variation in values among older participants. Assays for plasma human Aβ_40_, Aβ_42_, total tau, and neurofilament light chain (NfL) were performed on a single molecule array HD‐1 analyzer platform (Human Neurology 3‐Plex A assay for Aβ_40_, Aβ_42_, and total tau; Nf‐light advantage kit for NfL). All samples were assayed in duplicate and were run with kits from the same lot for each analyte. The median coefficients of variation by assay batch were < 10% for Aβ_40_, Aβ_42_, and total tau, and between 10 and 20% for NfL.

### 2.5 Cognitive assessments

All participants underwent a cognitive test battery assessing memory, processing speed, language, and executive function,^35^ including the Montreal Cognitive Assessment (MoCA). Testing occurred three to four times over the course of the study: at screening or randomization visit, at 2‐year and 4‐year visits, and at study close‐out if it was > 1 year after the 4‐year follow‐up visit. An expert adjudication panel of neurologists, neuropsychologists, geriatricians, and geropsychologists reviewed participants’ cognitive scores, functional status assessment, and demographic and medical history and classified the participants into three primary categories at each assessment visit: no cognitive impairment, MCI, or probable dementia.^3^ Procedures for adjudicating MCI and dementia are described in the study protocol.^3^ Only participants with two consecutive expert classifications of MCI were defined as having MCI.^28^ Time to the first of two consecutive occurrences of MCI was used as the time to outcome for MCI. Time to the first identification of adjudicated dementia was used as the time to outcome for probable dementia (PD).^3^, ^28^

### 2.6 Statistical analyses

All statistical analyses were conducted in Stata (v17). Distributions of all variables of interest and covariates were inspected visually. Demographic and lifestyle differences between participants with no adjudication with MCI or PD and with adjudication with MCI were tested using a Student *t* test. Pearson's correlation matrix and the corresponding *P* values were used to quantify the relationships between the a priori selected ROI volumes and CVRs. Age at randomization (and MRI scan), sex, education, race, regional volumes, systolic and diastolic blood pressure measures, heart rate, and total brain white matter lesions were treated as potential confounders in our analyses. Confounding was established if adding the candidate variable to the model changed the coefficient of regional CVR by > 20%. All potential confounders met this criterion for confounding and were included in the analyses as covariates.

Cox proportional hazards models were used to assess the relationship between baseline regional CVR and MCI and dementia. The time since randomization to the adjudication of MCI or dementia was used in analyses. Visual inspection of Martingale residuals plotted against variables of interest was used to assess linearity. The correlation of follow‐up time with Schoenfeld residuals was used to assess if proportional hazards assumption was violated. Any variables violating linearity or proportional hazards assumptions would be transformed to meet these assumptions.

Interactions with treatment assignments were used to test whether high intensity blood pressure control modified the association of baseline regional CVR on MCI, dementia, and combined MCI/dementia outcomes. CVR–race interactions were used to test whether race modifies the association of baseline regional CVR and MCI risk, dementia, and the composite MCI/dementia outcome. These analyses focused on differences between participants identifying as non‐Hispanic White and non‐Hispanic Black. Modification by Hispanic ethnicity was not evaluated due to underrepresentation of Hispanic participants in the sample. Associations were considered significant if the two‐sided type 1 error rate was < 0.05. All association with regional CVR *P* values were adjusted using Benjamini–Hochberg procedure.

The analyses were repeated for participants with available plasma biomarkers. Aβ_42_/Aβ_40_, total tau, and NfL were treated as potential confounders. Additionally, E values^36^ for the estimates and for the confidence limits are presented as measures of sensitivity to potential unmeasured confounding for all analyses.

## 3 RESULTS

### 3.1 Baseline participant characteristics

**FIGURE 1:**
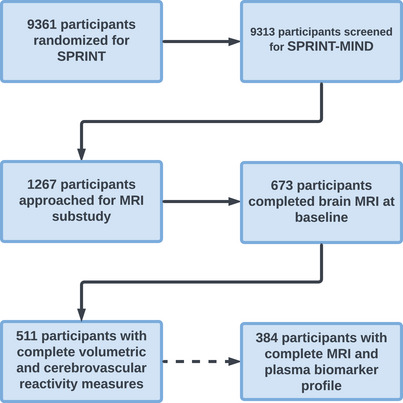
Flow diagram of study sample selection. MRI, magnetic resonance imaging; SPRINT‐MIND, Systolic Blood Pressure Intervention Trial Memory and Cognition in Decreased Hypertension

Five hundred eleven participants were selected for the current analysis of 673 participants with MRI (selected from the total sample of 9361 participants based on criteria described in the protocol [Figure 1]). One hundred forty‐six participants had missing occipital pole CVR or failed quality control. Sixteen more participants had missing CVR values in other ROIs. Participants selected for the MRI substudy had few demographic differences from those who were not selected. Significant differences were observed for race, such that the MRI substudy had a lower proportion of Hispanic‐identifying participants (4.66% vs. 10.89%, *P* < 0.01). Additionally, participants in the MRI substudy had higher baseline MoCA scores (23.5 [standard deviation (SD) = 4.16] vs. 22.75 [SD = 4.35], *P* < 0.01).

**FIGURE 2:**
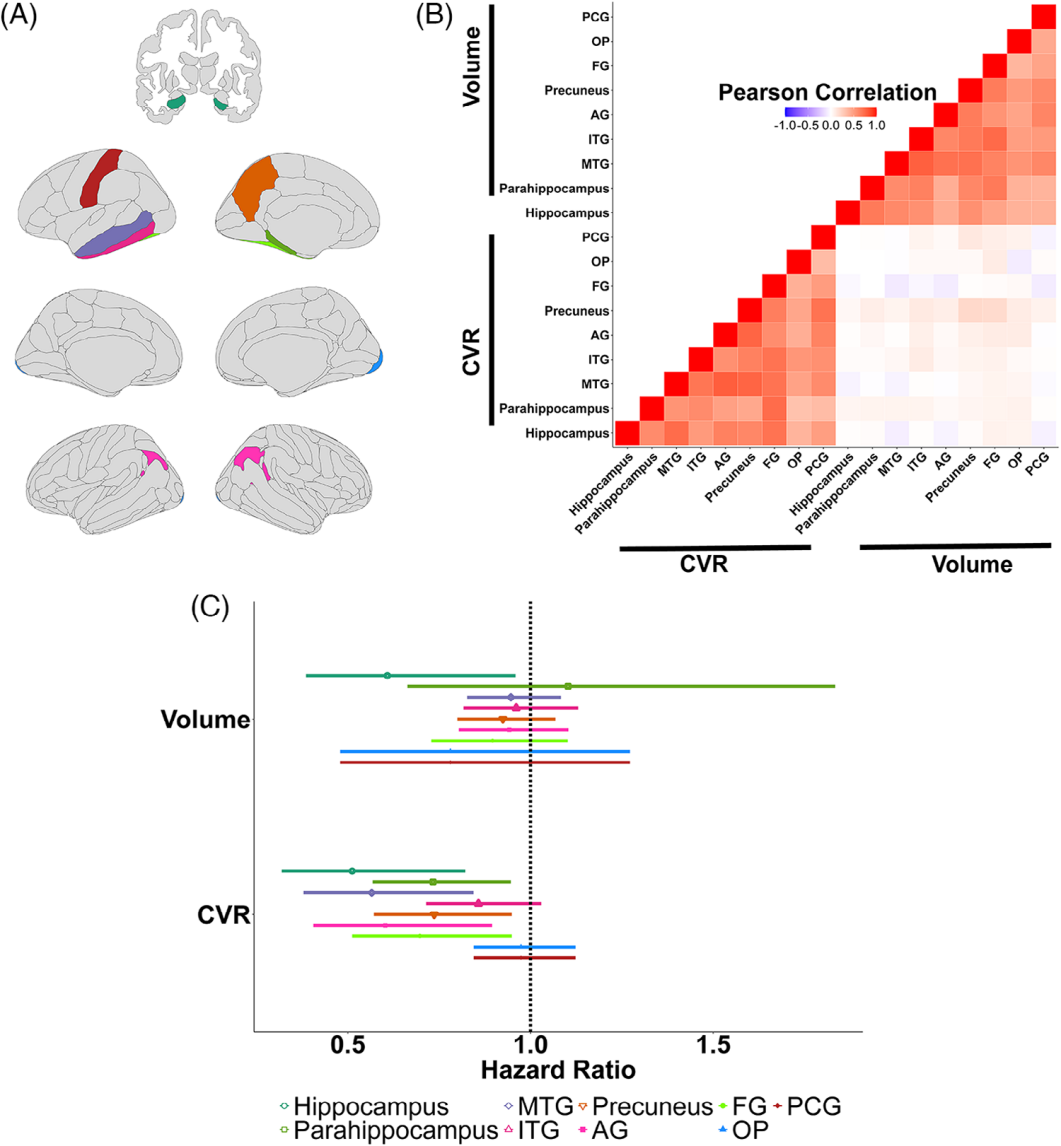
Relationships of regional CVR and volumes. A, Selected brain regions and control regions. B, Pearson correlations of regional volumes and CVRs with each other. C, Regional volume and CVR in the CVR–MCI hazard relationship (adjusted for age at randomization, treatment assignment, race, sex, and education). AG, angular gyrus; CVR, cerebrovascular reactivity; FG, fusiform gyrus; ITG, inferior temporal gyrus; MCI, mild cognitive impairment; MTG, middle temporal gyrus; OP, occipital pole; PCG, postcentral gyrus

Thirty‐four participants from the selected MRI cohort were adjudicated with MCI during the follow‐up period (median follow‐up = 5.36 years, interquartile range [IQR] = 2.12 years) and nine were adjudicated with possible dementia (median follow‐up = 5.5 years, IQR = 2.05). As shown in Table 1, compared to participants who remained cognitively normal, those adjudicated with MCI were significantly older (*P* = 0.01), had a higher proportion of people with high school degree or lower (*P* = 0.03), and had a significantly lower baseline MoCA score (*P* < 0.01). Significant differences in baseline CVR were observed for all AD signature regions, such that those who subsequently were adjudicated with MCI had a lower CVR in all regions. Additionally, significant baseline differences were observed for hippocampal volume (*P* = 0.02) between participants adjudicated with MCI and those that were cognitively normal during follow‐up. Each regional CVR measure was moderately to strongly correlated with other regional CVR measures; each regional volume was moderately to strongly correlated with other regional volumes (*r* > 0.42 for all regions, Figure 2A). Baseline CVR and volumes did not exhibit strong correlations (Figure 2B).

**TABLE 1:** Participant demographic, cognitive, and anatomical characteristics. Participants were compared using the Student *t* test for continuous variables and the chi‐square test for equal proportions for categorical variables. Bilateral regional measures are presented. Cohen *D* was used to assess the effect size of continuous variable comparisons and Cramer *V* was used for the categorical variables.

Variable	All	No adjudication with MCI	Adjudication with MCI	Effect size	*P* value
*N*	511	477	34	–	–
**Demographic variables**					
Randomization (% intensive treatment)	53.23	52.89	70.59	0.09	0.05
Sex (% male)	61.65	62.19	64.71	0.01	0.77
Education (reference: at most high school)				0.12	0.03
% College	51.08	51.65	47.06		
% Graduate education	26.88	28.31	14.71		
MoCA	23.61 (4.02)	24.02 (3.60)	17.76 (5.05)	1.69	<0.01
Race (reference: non‐Hispanic White)				0.07	0.49
% Black	31.54	30.37	35.29		
% Hispanic	4.66	4.13	8.82		
Age at randomization (years)	67.39 (7.90)	67.14 (7.91)	71.00 (7.03)	0.49	0.01
Systolic blood pressure (mmHg)	145 (11.2)	145 (11.1)	149 (12.8)	0.38	0.07
Diastolic blood pressure (mmHg)	80.3 (11.6)	81.4 (11.4)	78.1 (12.4)	0.29	0.15
Heart rate (beats per minute)	68.3 (12.0)	67.9 (11.9)	66.4 (13.4)	0.13	0.337
Framingham risk score (%)	17.4 (2.50)	17.3 (2.42)	18.5 (2.35)	0.51	0.006
White matter lesion volume (cm^3^)	5.38 (6.42)	5.10 (6.14)	9.54 (8.71)	0.70	0.008
**CVR measures (% change)**					
Hippocampus	2.01 (0.89)	2.04 (0.90)	1.61 (0.61)	0.49	0.01
Parahippocampus	3.74 (1.75)	3.79 (1.76)	3.01 (1.52)	0.45	0.01
Middle temporal gyrus	2.59 (0.99)	2.63 (1.00)	2.10 (0.75)	0.54	<0.01
Inferior temporal gyrus	4.81 (1.99)	4.85 (2.00)	4.14 (1.80)	0.36	0.05
Precuneus	3.37 (1.52)	3.41 (1.54)	2.73 (1.03)	0.45	0.01
Angular gyrus	2.34 (0.95)	2.36 (0.95)	1.98 (0.78)	0.4	0.03
Fusiform gyrus	3.21 (1.32)	3.25 (1.33)	2.65 (0.99)	0.46	0.01
Occipital pole	4.52(2.64)	4.55 (2.66)	4.16 (2.20)	0.15	0.43
Postcentral gyrus	2.65 (1.24)	2.67 (1.28)	2.45(0.75)	0.17	0.1
**Volumes (cm^3^)**					
Hippocampus	7.57 (0.80)	7.59 (0.80)	7.26 (0.81)	0.42	0.02
Parahippocampus	7.41 (0.92)	7.41 (0.92)	7.35 (0.86)	0.07	0.71
Middle temporal gyrus	24.14 (3.28)	24.20 (3.31)	23.29 (2.75)	0.28	0.12
Inferior temporal gyrus	21.76 (2.85)	21.80 (2.85)	21.23 (2.85)	0.2	0.26
Precuneus	21.34 (3.34)	21.41 (3.31)	20.33 (3.59)	0.32	0.07
Angular gyrus	16.74 (2.49)	16.79 (2.51)	16.00 (2.14)	0.32	0.07
Fusiform gyrus	16.23 (2.33)	15.54 (2.10)	16.28 (2.34)	0.32	0.07
Occipital pole	5.11 (0.88)	5.12 (0.89)	4.92 (0.72)	0.24	0.18
Postcentral gyrus	17.31 (2.31)	17.38 (2.36)	16.89 (1.91)	0.21	0.14

Abbreviations: MCI, mild cognitive impairment; MoCA, Montreal Cognitive Assessment.

Three hundred eighty‐four participants had full plasma biomarker profiles (31 of these were adjudicated with MCI). Mean Aβ_42_/Aβ_40_ was 0.16 (SD = 0.36), mean total tau was 8.43 pg/mL (SD = 3.82), and mean NfL was 17.43 pg/mL (SD = 14.91). Plasma biomarker levels did not significantly differ for any of the biomarkers (*P* = 0.52 for Aβ_42_/Aβ_40_, *P* = 0.84 for total tau, and *P* = 0.72 for NfL).

Baseline systolic blood pressure was moderately correlated with regional CVRs (all *r*s ≤ 0.18). Baseline systolic and diastolic blood pressures, baseline heart rate, and total brain white matter lesions were not found to confound the relationship between regional CVR and MCI risk.

### 3.2 Regional CVR in AD signature regions is inversely associated with MCI risk

After adjusting for confounding by regional volumes, significant inverse regional CVR–MCI risk associations were observed for hippocampus (*P* = 0.02), parahippocampus (*P* = 0.03), MTG (*P* = 0.02), precuneus (P = 0.03), AG (P = 0.03), and FG (P = 0.03). No significant ITG CVR–MCI risk association was observed (*P* = 0.11; Table 2, Figure 1C). In the same models, only the hippocampal volume was significantly inversely associated with MCI risk (*P* = 0.04). There was no significant OP CVR–MCI risk association and a non‐significant trend level PCG CVR–MCI risk association was observed (*P* = 0.72 and *P* = 0.10, respectively). All models were additionally adjusted for age at randomization, sex, education, treatment assignment, and race/ethnicity. Regional volume‐weighted AD signature composite CVR was negatively associated with adjudication with MCI in the follow‐up (hazard ratio [HR] = 0.61 per 1 unit change, 95% confidence interval [CI]: [0.43, 0.85], *P* = 0.02) after adjusting for these covariates.

**TABLE 2:** Cox regression hazard rate ratio estimates and 95% confidence intervals (adjudication of MCI as the outcome). College educated non‐Hispanic White females randomized into low intensity treatment arm were treated as the reference group. Regional volume for MTG, ITG, and precuneus were transformed (raised to the power of −2 and multiplied by −1) to meet the assumption of proportional hazards.

	Region model				
Variable	Hippocampus	Parahippocampus	MTG	ITG	Precuneus
Randomization (intensive treatment)	2.21 (1.04, 4.71)	2.31 (1.09, 4.93)	2.37 (1.11, 5.05)	2.39 (1.12, 5.08)	2.54 (1.20, 5.38)
Age at randomization	1.08 (1.03, 1.13)	1.10 (1.05, 1.15)	1.09 (1.04, 1.14)	1.09 (1.04, 1.14)	1.09 (1.04, 1.14)
Sex (male)	2.02 (1.39, 18.63)	1.50 (0.62, 3.64)	2.10 (0.94, 4.72)	1.98 (0.83, 4.69)	2.23 (0.99, 5.06)
Education					
Graduate	0.56 (0.20, 1.56)	0.52 (0.19, 1.44)	0.50 (0.18, 1.37)	0.49 (0.18, 1.36)	0.51 (0.18, 1.43)
At most high school	2.10 (0.99, 4.47)	2.40 (1.12, 5.13)	2.30 (1.09, 4.88)	2.43 (1.15, 5.14)	2.80 (1.34, 5.84)
Race					
Black	1.67 (0.75, 3.71)	1.94 (0.82, 4.58)	2.07 (0.86, 4.97)	1.83 (0.80, 4.19)	1.60 (0.70, 3.65)
Hispanic	5.08 (1.39, 18.63)	5.39 (1.41, 20.54)	4.88 (1.3, 18.29)	4.93 (1.30, 18.78)	4.83 (1.28, 18.22)
Region volume	0.61 (0.39, 0.96)	1.10 (0.66, 1.83)	0.95 (0.83, 1.08)	0.96 (0.82, 1.13)	0.92 (0.80, 1.07)
Region CVR	0.51 (0.32, 0.82)	0.73 (0.56, 0.95)	0.57 (0.38, 0.85)	0.86 (0.71, 1.03)	0.74 (0.57, 0.95)

	AG	FG	OP	PCG	AD composite
Randomization (intensive treatment)	2.71 (1.28, 5.73)	2.02 (0.94, 4.32)	2.02 (0.93, 4.38)	2.67 (1.25, 5.69)	2.40 (1.12, 5.11)
Age at randomization	1.09 (1.04, 1.15)	1.09 (1.04, 1.14)	1.08 (1.03, 1.14)	1.10 (1.05, 1.16)	1.10 (1.05, 1.15)
Sex (male)	1.85 (0.88, 3.89)	2.15 (0.92, 5.05)	1.61 (0.72, 3.61)	1.80 (0.85, 3.82)	1.61 (0.77, 3.36)
Education					
Graduate	0.51 (0.19, 1.42)	0.55 (0.20, 1.55)	0.53 (0.19, 1.50)	0.48 (0.17, 1.34)	0.54 (0.19, 1.50)
At most high school	2.81 (1.35, 5.87)	2.25 (1.05, 4.82)	2.13 (0.94, 4.81)	2.85 (1.34, 6.07)	2.30 (1.07, 4.92)
Race					
Black	1.93 (0.87, 4.27)	1.73 (0.74, 4.03)	1.94 (0.85, 4.44)	1.86 (0.82, 4.20)	2.01 (0.89, 4.57)
Hispanic	5.40 (1.45, 20.09)	5.42 (1.45, 20.21)	5.64 (1.46, 21.75)	6.20 (1.59, 24.25)	5.21 (1.38, 19.62)
Region volume	0.94 (0.80, 1.10)	0.90 (0.73, 1.10)	0.78 (0.48, 1.27)	1.02 (0.86, 1.21)	–
Region CVR	0.60 (0.41, 0.89)	0.70 (0.51, 0.95)	0.97 (0.84, 1.12)	0.76 (0.55, 1.03)	0.61 (0.43, 0.85)

Abbreviations: AD, Alzheimer's disease; AG, angular gyrus; CVR, cerebrovascular reactivity; FG, fusiform gyrus; ITG, inferior temporal gyrus; MCI, mild cognitive impairment; MTG, middle temporal gyrus; OP, occipital pole; PCG, postcentral gyrus.

### 3.3 Regional CVR in AD signature regions is inversely associated with MCI risk, adjusted for plasma AD biomarkers

After adjusting for confounding by regional volumes and plasma Aβ_42_/Aβ_40_, total tau, and NfL, significance inverse regional CVR–MCI risk associations were observed for hippocampus (*P* = 0.01; Table 3), parahippocampus (*P* = 0.02), MTG (*P* = 0.01), ITG (*P* = 0.048), precuneus (*P* = 0.02), AG (*P* = 0.02), FG (*P* = 0.02), and AD signature composite CVR (*P* = 0.01). There were no significant OP CVR–MCI risk or PCG CVR–MCI risk associations (*P* = 0.53, and *P* = 0.052, respectively; Table 3). All analyses were controlled for false discovery rate.

**TABLE 3:** Cox regression hazard rate ratio estimates and 95% confidence intervals (adjudication of MCI as the outcome, additionally adjusting for plasma biomarkers). College educated non‐Hispanic White females randomized into low intensity treatment arm were treated as the reference group.

	Region model				
Variable	Hippocampus	Parahippocampus	MTG	ITG	Precuneus
Randomization (intensive treatment)	2.16 (0.97, 4.77)	2.4 (1.07, 5.41)	2.24 (1.00, 5.05)	2.29 (1.02, 5.12)	2.46 (1.10, 5.46)
Age at randomization	1.08 (1.01, 1.15)	1.08 (1.02, 1.14)	1.08 (1.01, 1.15)	1.08 (1.01, 1.14)	1.08 (1.01, 1.15)
Sex (male)	1.64 (0.72, 3.66)	1.28 (0.52, 3.2)	1.72 (0.73, 4.03)	1.62 (0.63, 4.15)	2.02 (0.83, 4.89)
Education					
Graduate	0.47 (0.15, 1.46)	0.44 (0.14, 1.35)	0.46 (0.15, 1.43)	0.44 (0.14, 1.35)	0.49 (0.16, 1.54)
At most high school	2.06 (0.90, 4.71)	2.62 (1.15, 5.99)	2.17 (0.95, 4.98)	2.23 (0.97, 5.13)	2.69 (1.19, 6.09)
Race					
Black	1.56 (0.68, 3.72)	1.85 (0.76, 4.53)	2.01 (0.81, 4.95)	1.64 (0.69, 3.90)	1.60 (0.67, 3.84)
Hispanic	5.15 (1.04, 25.50)	6.90 (1.37, 34.64)	5.13 (0.98, 26.83)	5.81 (1.10, 30.74)	5.41 (1.09, 26.94)
Aβ_42_/Aβ_40_	0.05 (0.0002, 10.94)	0.08 (0.0004, 14.23)	0.06 (0.0003, 12.6)	0.07 (0.0003, 13.69)	0.07 (0.004, 12.74)
Total tau	1.03 (0.93, 1.15)	1.04 (0.93, 1.17)	1.05 (0.93, 1.17)	1.04 (0.94, 1.17)	1.03 (0.92, 1.15)
Neurofilament light chain	0.97 (0.93, 1.01)	0.98 (0.95, 1.02)	0.98 (0.95, 1.02)	0.98 (0.94, 1.02)	0.98 (0.94, 1.02)
Region volume	0.66 (0.41, 1.06)	1.12 (0.66, 1.89)	0.95 (0.82, 1.11)	0.97 (0.81, 1.16)	0.91 (0.78, 1.06)
Region CVR	0.43 (0.25, 0.75)	0.71 (0.54, 0.93)	0.47 (0.3, 0.75)	0.81 (0.66, 0.99)	0.70 (0.52, 0.93)

	AG	FG	OP	PCG	AD composite
Randomization (intensive treatment)	2.63 (1.18, 5.88)	1.98 (0.88, 4.47)	2.08 (0.91, 4.75)	2.65 (1.17, 5.99)	2.40 (1.06, 5.4)
Age at randomization	1.08 (1.02, 1.15)	1.08 (1.02, 1.15)	1.07 (1.01, 1.13)	1.09 (1.02, 1.16)	1.09 (1.02, 1.15)
Sex (male)	1.54 (0.69, 3.43)	1.71 (0.69, 4.23)	1.57 (0.66, 3.71)	1.53 (0.69, 3.38)	1.39 (0.64, 3.03)
Education					
Graduate	0.44 (0.14, 1.37)	0.45 (0.15, 1.41)	0.45 (0.14, 1.42)	0.40 (0.13, 1.24)	0.47 (0.15, 1.45)
At most high school	2.66 (1.19, 5.96)	2.25 (0.98, 5.14)	2.21 (0.93, 5.24)	2.69 (1.16, 6.2)	2.50 (1.09, 5.75)
Race					
Black	1.96 (0.84, 4.57)	1.64 (0.67, 4.01)	1.88 (0.79, 4.46)	1.96 (0.83, 4.61)	1.92 (0.82, 4.54)
Hispanic	6.06 (1.20, 30.66)	5.99 (1.18, 30.42)	5.71 (1.10, 29.73)	7.23 (1.32, 39.54)	6.05 (1.2, 30.5)
Aβ_42_/Aβ_40_	0.14 (0.0009, 20.7)	0.05 (0.0002, 10.31)	0.02 (0.00005, 8.52)	0.06 (0.003, 11.48)	0.06 (0.003, 13.40)
Total tau	1.03 (0.92, 1.15)	1.05 (0.94, 1.17)	1.03 (0.92, 1.15)	1.02 (0.91, 1.14)	1.04 (0.93, 1.16)
Neurofilament light chain	0.98 (0.94, 1.02)	0.98 (0.94, 1.01)	0.98 (0.95, 1.02)	0.98 (0.94, 1.02)	0.98 (0.94, 1.02)
Region volume	0.95 (0.8, 1.13)	0.93 (0.76, 1.15)	0.74 (0.45, 1.23)	1.04 (0.87, 1.24)	–
Region CVR	0.58 (0.38, 0.88)	0.61 (0.43, 0.87)	0.95 (0.82, 1.11)	0.68 (0.47, 1.00)	0.55 (0.38, 0.81)

Abbreviations: Aβ, amyloid beta; AD, Alzheimer's disease; AG, angular gyrus; CVR, cerebrovascular reactivity; FG, fusiform gyrus; ITG, inferior temporal gyrus; MCI, mild cognitive impairment; MTG, middle temporal gyrus; OP, occipital pole; PCG, postcentral gyrus.

As an estimate of unmeasured confounding that might explain these observed CVR–MCI risk associations, the minimum E value for the point estimate of these associations without adjustment for AD plasma biomarkers was 2.04 (the minimum E value for lower confidence interval value = 1.29) among statistically significant associations. After adjusting for plasma biomarkers, these numbers were 1.77 and 1.11, respectively (Table 4). Unmeasured confounding of these strengths would not suffice to explain away the effect estimates.

**TABLE 4:** E values for point estimates and the lower bound of confidence interval needed to explain the observed associations.

	Without adjustment for plasma biomarkers		With adjustment for plasma biomarkers	
Region	E value for point estimate	E value for confidence interval	E value for point estimate	E value for confidence interval
Hippocampus	3.33	1.74	4.08	2
Parahippocampus	2.08	1.29	2.17	1.36
MTG	2.9	1.63	3.68	2
ITG	1.6	1	1.77	1.11
Precuneus	2.04	1.29	2.21	1.36
AG	2.72	1.5	2.84	1.53
FG	2.21	1.29	2.66	1.56
OP	1.21	1.12	1.29	1
PCG	1.96	1	2.3	1
AD composite	2.66	1.63	3.04	1.77

Abbreviations: AD, Alzheimer's disease; AG, angular gyrus; FG, fusiform gyrus; ITG, inferior temporal gyrus; MTG, middle temporal gyrus; OP, occipital pole; PCG, postcentral gyrus.

### 3.4 Regional CVR in AD signature regions is not associated with combined dementia and MCI risk

After controlling for false discovery rate using Benjamini–Hochberg procedure and age at randomization, sex, education, treatment assignment, race/ethnicity, and confounding by regional volumes, no significant association between regional CVR and the composite outcome of MCI and/or probable dementia was observed (*P* > 0.16 for all regions; Table 5).

**TABLE 5:** Cox regression hazard rate ratio estimates and 95% confidence intervals (adjudication of MCI or dementia as the outcome). College educated non‐Hispanic White females randomized into low intensity treatment arm were treated as the reference group. Regional volume for MTG, ITG, and precuneus were transformed (raised to the power of −2 and multiplied by −1) to meet the proportional hazards assumption.

	Region model				
Variable	Hippocampus	Parahippocampus	MTG	ITG	Precuneus
Randomization (Intensive treatment)	2.12 (1.08, 4.15)	2.16 (1.10, 4.24)	2.28 (1.17, 4.46)	2.29 (1.18, 4.47)	2.43 (1.25, 4.72)
Age at randomization	1.09 (1.05, 1.14)	1.10 (1.06, 1.15)	1.09 (1.04, 1.14)	1.10 (1.06, 1.14)	1.10 (1.05, 1.15)
Sex (male)	1.57 (0.79, 3.14)	1.16 (0.52, 2.55)	1.52 (0.75, 3.08)	1.46 (0.68, 3.15)	1.43 (0.70, 2.93)
Education					
Graduate	0.66 (0.27, 1.60)	0.60 (0.25, 1.45)	0.57 (0.24, 1.37)	1.46 (0.68, 3.15)	0.59 (0.25, 1.43)
At most high school	2.06 (1.02, 4.16)	2.22 (1.09, 4.52)	2.02 (1.01, 4.04)	2.16 (1.08, 4.33)	2.51 (1.27, 4.96)
Race					
Black	1.76(0.85, 3.65)	1.98 (1.10, 4.24)	1.78 (0.84, 3.78)	1.83 (0.89, 3.75)	1.89 (0.91, 3.92)
Hispanic	4.67 (1.31, 16.67)	5.13 (1.41, 18.60)	4.41 (1.22, 15.92)	4.85(1.33, 17.62)	4.79 (1.32, 17.37)
Region volume	0.59 (0.38, 0.91)	1.03 (0.64, 1.64)	0.93 (0.82, 1.05)	0.94 (0.81, 1.09)	0.94 (0.83, 1.07)
Region CVR	0.66 (0.44, 0.98)	0.90 (0.74, 1.10)	0.72 (0.52, 1.002)	0.95 (0.82, 1.10)	0.88 (0.72, 1.08)

	AG	FG	OP	PCG	AD composite
Randomization (intensive treatment)	2.37 (1.21, 4.64)	2.05 (1.05, 4.02)	1.92 (0.97, 3.82)	2.41 (1.23, 4.73)	2.25 (1.15, 4.43)
Age at randomization	1.10 (1.05, 1.15)	1.10 (1.06, 1.15)	1.10 (1.05, 1.14)	1.11 (1.06, 1.16)	1.11 (1.06, 1.15)
Sex (male)	1.38 (0.71, 2.70)	1.30 (0.62, 2.73)	1.13 (0.56, 2.29	1.21 (0.63, 2.32)	1.16 (0.61, 2.20)
Education					
Graduate	0.60 (0.25, 1.44)	0.61 (0.25, 1.47)	0.61 (0.25, 1.48)	0.56 (0.23, 1.35)	0.61 (0.25, 1.49)
At most high school	2.54 (1.27, 5.05)	2.02 (1.00, 4.10)	1.96 (0.92, 4.17)	2.41 (1.20, 4.84)	2.19 (1.07, 4.46)
Race					
Black	1.93 (0.94, 3.93)	2.11 (1.01, 4.41)	2.14 (1.04, 4.41)	2.13 (1.04, 4.36)	2.10 (1.02, 4.35)
Hispanic	4.79 (1.33, 17.20)	5.33 (1.48, 19.19)	5.54 (1.48, 20.68)	2.41 (1.20, 4.84)	5.13 (1.43, 18.48)
Region volume	0.93 (0.80, 1.08)	0.96 (0.80, 1.15)	0.82 (0.54, 1.26)	1.02 (0.88, 1.19)	–
Region CVR	0.80 (0.57, 1.10)	0.78 (0.61, 1.01)	1.02 (0.90, 1.15)	0.89 (0.71, 1.12)	0.79 (0.60, 1.04)

Abbreviations: AD, Alzheimer's disease; AG, angular gyrus; CVR, cerebrovascular reactivity; FG, fusiform gyrus; ITG, inferior temporal gyrus; MCI, mild cognitive impairment; MTG, middle temporal gyrus; OP, occipital pole; PCG, postcentral gyrus.

### 3.5 Effect modification by treatment assignment and race

No significant randomization by regional CVR interactions was observed for any AD region of interest (all interaction *P* values > 0.45). Black versus White race did not significantly modify the association of regional CVR on MCI risk (all interaction *P* values > 0.10).

## 4 DISCUSSION

In the current study we found that higher CVR in AD signature regions was associated with lower MCI risk. These findings implicate lower CVR as a biomarker of cognitive decline and conversion to MCI, identifying an early, non‐invasive MRI technique for indicating risk of future cognitive decline. The current study found statistically significant associations between AD signature region CVRs and MCI risk, while controlling for regional brain volume, an established indicator of dementia risk,^24^, ^25^, ^26^ as a confounder. Additionally, CVR was a stronger predictor of MCI than regional brain volumes, indicating that CVR may be an earlier predictor of cognitive decline compared to brain volume/atrophy. These relationships were not observed for CVR in the OP and PCG, suggesting that the relationship is primarily apparent in AD signature regions. CVR associations were also not found for the composite of MCI or probable dementia as an outcome, possibly suggesting that CVR is more associated with earlier stages of decline. The associations were robust. Any unmeasured confounder had to be associated with at least a 1.77‐fold increase in MCI risk and standardized effect size change of 1.77 on CVR to explain away the observed relationships.

Cerebrovascular dysfunction is among the key pathologic factors of AD and dementia.^37^ Studies have established that dysfunction characterized by blood–brain barrier (BBB) breakdown and cerebral blood flow (CBF) reduction contribute to AD.^38^ CBF dysregulation and reduction is known to be associated with increased Aβ production.^39^ However, BBB imaging studies have shown that older adults with cognitive dysfunction have BBB breakdown regardless of amyloid/tau biomarker changes,^40^, ^41^, ^42^ suggesting that CVR reduction may be a contributor to cognitive decline due to cerebrovascular disease that may or may not interact with AD pathologies. Additionally, in a large population study, cerebral hypoperfusion (measured through CBF) preceded the onset of clinical dementia.^43^ Impaired CVR indicates loss of cerebrovascular integrity, accompanied with CBF reduction and BBB function disruptions.^39^ Given recent methodological advancements in CVR mapping using MRI,^44^ wider adoption of CVR mapping might help elucidate underlying vascular mechanisms of dementia pathology.

Although higher CVR in the AD signature regions that typically thin or atrophy throughout AD progression is associated with lower MCI risk,^24^, ^25^, ^26^ regional volumes other than the hippocampus were not associated with MCI risk, indicating that CVR changes may precede morphometric changes, consistent with other studies suggesting BBB breakdown and CBF dysregulation precede neurodegeneration.^39^, ^40^ These findings corroborate cross‐sectional human studies and rodent models, which correlate CVR reduction with AD progression.^6^, ^7^, ^8^, ^9^, ^10^, ^12^, ^13^, ^14^, ^15^, ^16^, ^22^, ^23^

Human studies have shown that participants with MCI and dementia have lower CVR compared to cognitively unimpaired individuals.^6^, ^8^, ^10^ A small pilot study identified that participants with preclinical and clinical AD had lower CVR, measured by a breath‐holding index and a breath‐holding acceleration index, compared to cognitively healthy participants.^6^ Another study found that cognitive decline in amnestic MCI and AD, assessed by the Mini‐Mental State Examination (MMSE) score for global cognition, was correlated with reduced CVR velocity, and that the change in velocity was not related to general or focal gray matter atrophy, microangiopathy, or motion.^8^ In another study, CVR in both gray matter and white matter of the whole brain was positively associated with cognitive performance, assessed with MMSE, among cognitively normal participants and participants with MCI.^10^ These findings are aligned with other imaging efforts to relate CVR to the default mode network, a network implicated in AD progression,^23^ among a cohort of younger subjects, indicating that CVR‐related changes in cognition may be independent from the progression of AD‐related pathologies. The present study identifies an association between regional CVR and MCI, suggesting a possible protective effect of CVR in key AD brain regions. This finding opens the possibility that maintaining higher CVR may lower the risk of conversion to MCI, and subsequently to dementia although the present study was not powered to examine the association with incident dementia.

Participants for the current study were selected from the SPRINT‐MIND trial, which evaluated the effect of intensive blood pressure control on incident dementia. It was important to assess whether the treatment modified the findings of this study. The relationship between CVR and MCI risk was not modified by treatment group (intensive vs. standard therapy). This could be due to differing underlying mechanisms through which CVR and blood pressure influence cognition or MCI, due to insufficient power to detect such an effect, or because the treatment may not have been of sufficient duration to observe a modifying effect.

Testing if race modifies the effect of CVR on adjudication with MCI is important. While cardiovascular diseases are more prevalent among non‐Hispanic Black individuals,^45^ research is needed to understand racial differences in cerebrovascular health. Although we may be underpowered to detect such differences, we did not find any significant racial/ethnic differences in the relationship between CVR and MCI risk.

## 5 LIMITATIONS

Although the data used in this study come from a clinical trial, the MRI substudy participants were a non‐random subgroup of the overall trial population. The data analyzed were observational, such that no directed modification to CVR was conducted in the context of a clinical trial or intervention. This approach limits conclusions of causality of CVR effects on MCI. Additionally, this observational study used CVR at the baseline only and thus the combined effect of treatment and CVR could not be evaluated. The small number of cases of probable dementia (*n* = 9) in the sample limits our ability to study the relationship between CVR and dementia risk. Additionally, the etiology of MCI and dementia was not determined, and there is a possibility that the CVR–MCI relationship is driven by other causes of dementia.

To minimize participant burden, the CVR measurement protocol did not involve measuring end tidal CO_2_ and the study did not have extrinsic measures of compliance. These decisions may result in underestimated values for vascular reactivity. Some populations with respiratory illnesses may have more biased results from this technique; however, we do not expect our study population to have disproportionally affected results due to the protocol. Additionally, CVR using the breath‐hold technique was found to be correlated with controlled deep breathing CVR, with CO_2_ inhalation CVR, and with resting state fluctuation amplitude; comparing BOLD versus arterial spin labeling (ASL), studies have found that ASL had one third of the activation, and we conclude that our results should be reproducible with another CVR measuring technique.^46^

No Apolipoprotein E (*APOE*) genotyping was performed on the study sample; however, given the E values calculated for the associations, *APOE*4 carrier status had to increase the risk of MCI by at least 1.77 in the presence of the other AD biomarkers to explain away the observed associations. In the ROSMAP study, *APOE*4 was associated with a 1.25‐fold increase in risk of incident MCI (compared to *APOE* ε3/ε3, 95% CI: 1.05, 1.49).^47^ In a population‐based study set in rural China, *APOE*4 had an odds ratio of 1.14 (95% CI: 0.95, 1.36).^48^ *APOE*4 was also associated with 1.4‐fold increase in incident MCI risk among Black participants enrolled in National Alzheimer's Coordinating Center (95% CI: 1.10, 1.78).^49^ While there is no current meta‐analysis or population‐wide study restricting the estimate of the effect of *APOE*4 on incident MCI, we believe it is unlikely that the estimate will be greater than 1.77 after adjusting for age, sex, education, race, and AD biomarkers and explain away our effect estimates.

### 5.1 Future directions

The present study focused on AD signature regions due to AD being one possible etiology for dementia. Future studies should investigate whether CVR within regions affected in other causes for dementia (e.g., vascular dementia, Parkinson's disease, dementia with Lewy bodies, or frontotemporal dementia) is also related to cognitive impairment, and whether those associations are confounded by regional volumes. Previous studies focused on cross‐sectional differences in CVR between participants with different cognitive impairment levels and the role of default mode brain network in these associations was noted.^23^ Longitudinal studies may extend this finding by investigating the relationship between CVR in regions involved in the network and incident MCI and dementia. CVR changes may also affect cognitive impairment through other mechanisms (CAA, atherosclerosis, arteriolosclerosis,^5^ etc.), and future studies should investigate these mechanisms in studies in which more mechanism‐specific data are collected. Additionally, whole brain versus regional measures may have different findings. Future studies should investigate whether the CVR associations are specific to AD signature regions or whether whole brain CVR may be associated with lower risk for MCI and dementia.

## 6 CONCLUSIONS

The current study establishes a relationship between CVR in AD signature regions and cognitive decline, characterized by MCI, reaffirming the role of CVR and vascular dysfunction in dementia etiology. Identification and treatment of lower CVR might prevent or delay cognitive decline.

## CONFLICT OF INTEREST STATEMENT

The authors declare no conflicts of interest. Author disclosures are available in the supporting information.

## CONSENT STATEMENT

The SPRINT‐MIND study was approved by the institutional review board at each participating site, and each participant provided written informed consent. The present study did not require institutional review board approval.

## Supporting information

Supporting Information
